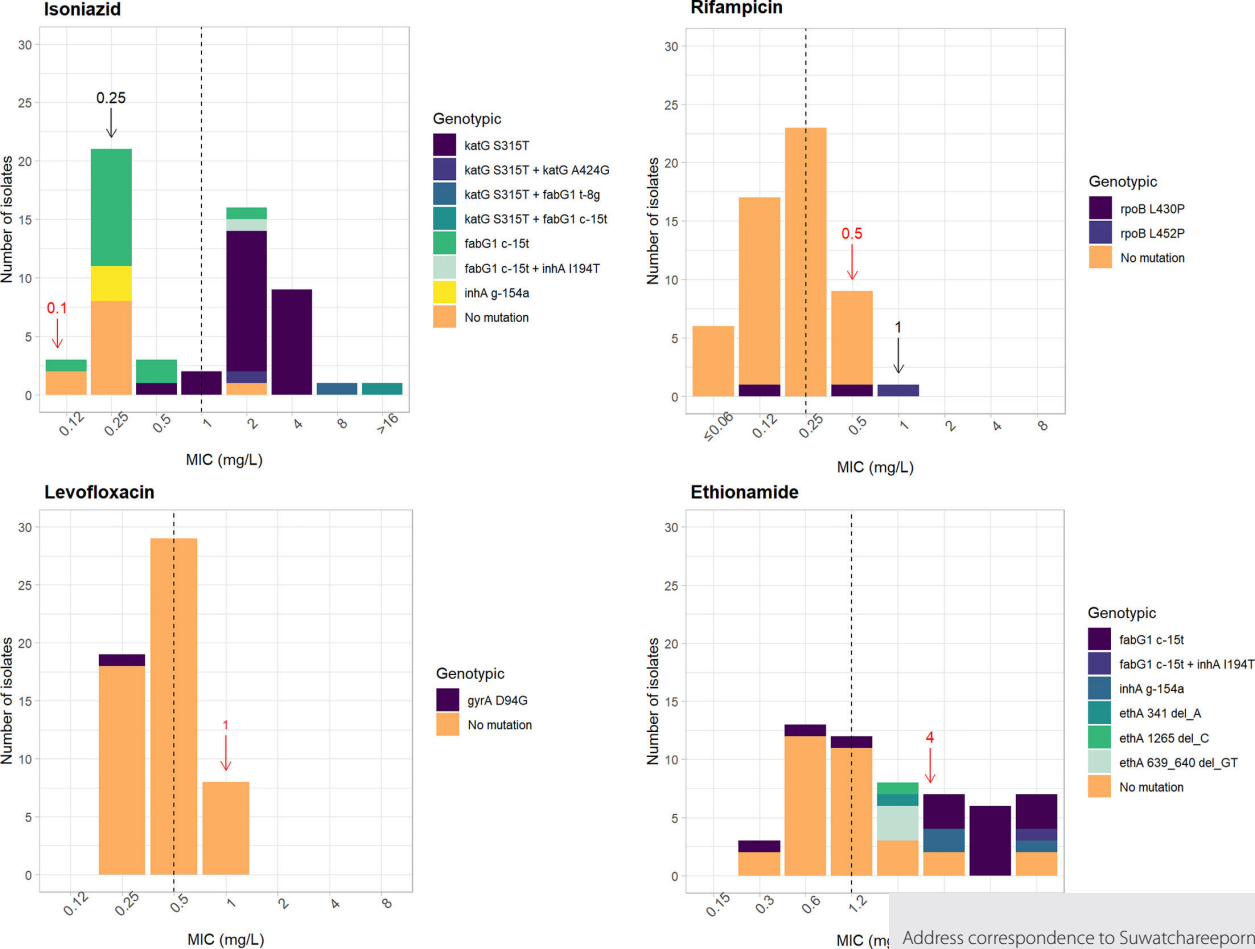# Erratum for Prommi et al., “Co-resistance to isoniazid and second-line anti-tuberculosis drugs in isoniazid-resistant tuberculosis at a tertiary care hospital in Thailand”

**DOI:** 10.1128/spectrum.02419-24

**Published:** 2024-11-14

**Authors:** Ajala Prommi, Kanphai Wongjarit, Suthidee Petsong, Ubonwan Somsukpiroh, Kiatichai Faksri, Kamon Kawkitinarong, Sunchai Payungporn, Suwatchareeporn Rotcheewaphan

## ERRATUM

Volume 12, no. 3, e03462-23, 2024, https://doi.org/10.1128/spectrum.03462-23. Top left of Fig. 1 should appear as shown in this erratum. The Isoniazid panel was omitted from the HTML version, although it does appear in the PDF version.

**Fig 1:**