# Supplementary material for: Co-resistance to isoniazid and second-line anti-tuberculosis drugs in isoniazid-resistant tuberculosis at a tertiary care hospital in Thailand
Source: Microbiol Spectr. 2024 Feb 7;12(3):e03462-23. doi: 10.1128/spectrum.03462-23 (PMC10913473; doi:10.1128/spectrum.03462-23)
Supplement: Fig. S1, Tables S1 to S6 — Supplemental figures and tables. [file spectrum.03462-23-s0001.pdf]

# Supplemental data

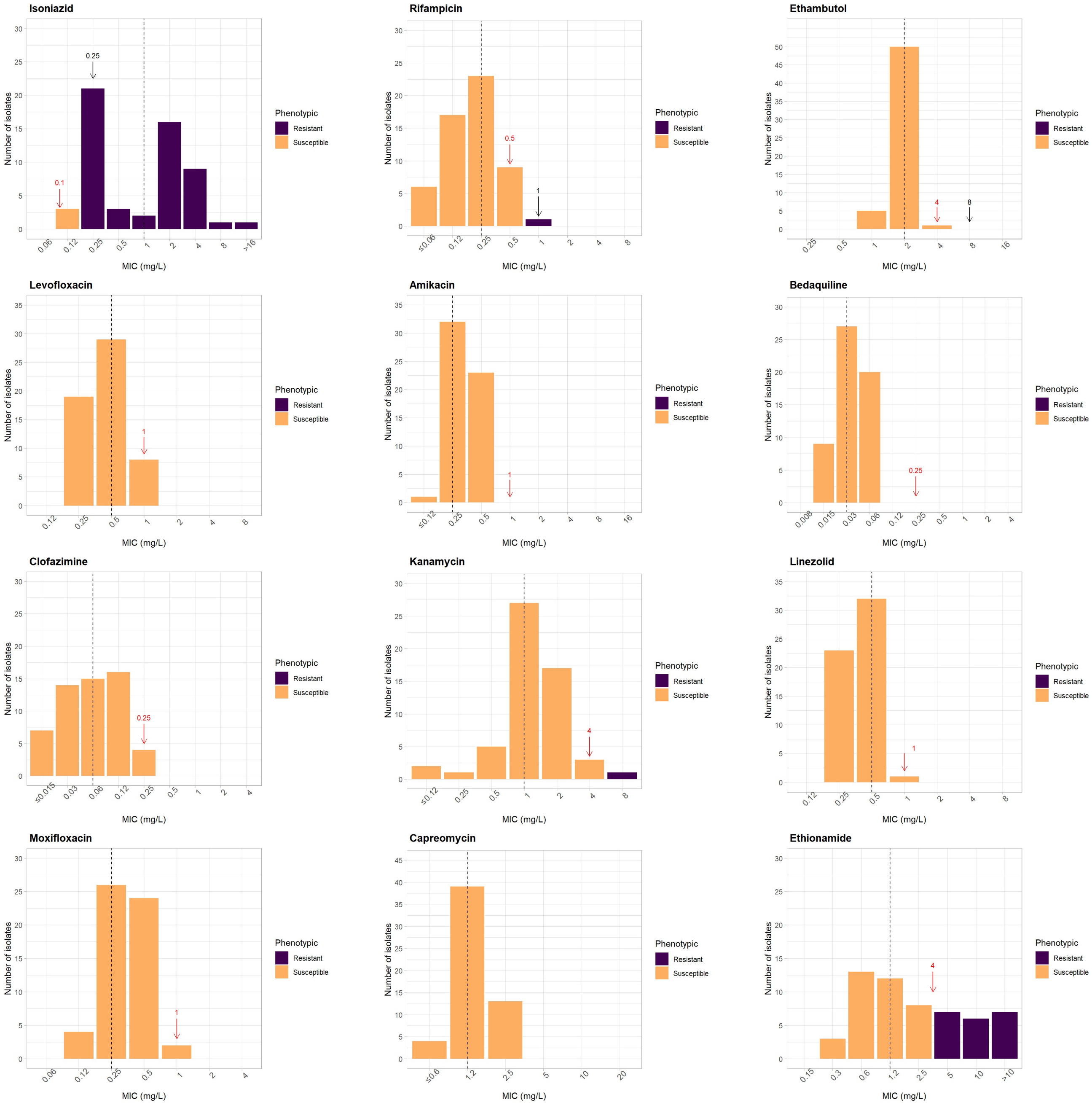


## Figure S1 MIC distributions of the 56 Hr-TB isolates in this study

The arrows indicate the ECOFF/ECVs (mg/L) of the CRyPTIC Consortium publication (red) (1) and the CLSI breakpoints (mg/L) (2) (black). The MIC cut-off value for capreomycin was not established. Dashed lines indicate the median MIC values of Hr-TB isolates (n = 56).

Abbreviations: MIC, minimal inhibitory concentration; Hr-TB, isoniazid-resistant tuberculosis; ECOFF/ECVs, epidemiological cut-off values.

**References**

1. The CRyPTIC Consortium. 2022. Epidemiological cut-off values for a 96-well broth microdilution plate for high-throughput research antibiotic susceptibility testing of *M. tuberculosis*. Eur Respir J 60:2200239.

2. CLSI. 2023. Performance standards for susceptibility testing of mycobacteria, *Nocardia* spp., and other aerobic Actinomycetes. In . CLSI Supplement M24S. Clinical and Laboratory Standards Institute.

**Table S1 The current critical concentrations (WHO), interpretative breakpoints (CLSI), and ECOFF/ECVs (CRyPTIC) for the MGIT and BMD methods of *M. tuberculosis* [e]**

| Drug | Concentration ranges (mg/L) of THAMYCO plate | WHO critical concentrations (mg/L) | CLSI MIC breakpoints (mg/L) [a] | | | CRyPTIC ECOFF/ECVs (mg/L) [a] |
|---|---|---|---|---|---|---|
| | | | S | Inconclusive | R | |
| RIF | 0.06 - 8 | 1 [b] | ≤ 0.5 | | ≥ 1 [c] | 0.5 |
| INH | 0.03 - 16 | 0.1 | ≤ 0.12 | | ≥ 0.25 | 0.1 |
| EMB | 0.25 - 16 | 5 | ≤ 2 | 4 | ≥ 8 | 4 |
| PZA [d] | | 100 | | | | |
| LFX | 0.12 - 8 | 1 | | | | 1 |
| MFX | 0.06 - 4 | 0.3 | | | | 1 |
| BDQ | 0.008 - 4 | 1 | | | | 0.25 |
| LZD | 0.12 - 8 | 1 | | | | 1 |
| CFZ | 0.015 - 4 | 1 | | | | 0.25 |
| DLM [d] | | 0.06 | | | | 0.25 |
| KAN | 0.12 - 16 | | | | | 4 |
| AMK | 0.12 - 16 | 1 | | | | 1 |
| ETO | 0.15 - 10 | 5 | | | | 4 |
| CAP | 0.6 - 20 | | | | | |

[a] 7H9 medium used for BMD was supplemented with 10% OADC and 0.5% glycerol.
[b] New critical concentration of RIF was endorsed by the WHO as 0.5 mg/L (1).
[c] New RIF breakpoint was endorsed by CLSI in 2023 (2).
[d] PZA and DLM were not tested by a broth microdilution method in this study.
[e] WHO, World Health Organization; CLSI, Clinical and Laboratory Standards Institute; ECOFF/ECVs, Epidemiological cut-off values; MTBC, *Mycobacterium tuberculosis* complex; MGIT, Mycobacterium Growth Indicator Tube; BMD, broth microdilution; MIC, minimal inhibitory concentration; RIF, rifampicin; INH, isoniazid; EMB, ethambutol; PZA, pyrazinamide; LFX, levofloxacin; MFX, moxifloxacin; BDQ, bedaquiline; LZD, linezolid; CFZ, clofazimine; DLM, delamanid; KAN, kanamycin; AMK, amikacin; ETO, ethionamide; CAP, capreomycin; R, resistant; S, susceptible.

## Reference


1. WHO. 2021. Technical report on critical concentrations for drug susceptibility testing of isoniazid and the rifamycins (rifampicin, rifabutin and rifapentine). Licence: CC BY-NC-SA 3.0 IGO. Geneva: World Health Organization.
2. CLSI. 2023. Performance standards for susceptibility testing of mycobacteria, *Nocardia* spp., and other aerobic actinomycetes. In . CLSI Supplement M24S. Clinical and Laboratory Standards Institute.

## Table S2 WGS-based gDST results from the TB-Profiler (No. = 56) [a, b]

| MTB clinical isolate | MTB SNP-based sublineage | Drug resistance | Genotypic drug susceptibility | Rifampicin (% frequency ) | Isoniazid (% frequency) | | Streptomycin (% frequency) | Ethionamide (% frequency) | | Fluoroquinolone (% frequency) |
|---|---|---|---|---|---|---|---|---|---|---|
| G1-1 | 2.2.1 | MDR-TB | RIF+INH R | *rpoB* L430P (100) | *katG* S315T (100) | | | | | |
| G1-2 | 2.2.1 | MDR-TB | RIF+INH R | *rpoB* L452P (100) | *katG* S315T (100) | | | | | |
| G1-3 | 2.2.1 | Other | FLQ+ETO R | | | | | *ethA* 341 del_A (100) | | *gyrA* D94G (100) |
| G1-4 | 2.2.1 | Hr-TB | INH R | | *katG* S315T (100) | | | | | |
| G2-1 | 1.2.1.2 | Hr-TB | INH R | | *katG* S315T (100) | | | | | |
| G2-4 | 2.2.1 | Hr-TB | INH R | | *katG* S315T (100) | | | | | |
| G2-5 | 1.1.3.3 | Hr-TB | INH+ETO R | | *katG* S315T (100) | *fabG1* c-15t (99) | | *fabG1* c-15t (99) | | |
| G2-6 | 1.2.1.2.1 | Hr-TB | INH+STM R | | *katG* S315T (99) | *fabG1* t-8g (99) | *gid* 102 del_G (100) | | | |
| G2-7 | 1.1.1 | Hr-TB | INH R | | *katG* S315T (100) | | | | | |
| G3-1 | 2.2.1 | Hr-TB | INH R | | *katG* S315T (100) | | | | | |
| G3-2 | 2.2.1 | Hr-TB | INH R | | *katG* S315T (100) | | | | | |
| G3-3 | 2.2.1 | Hr-TB | INH R | | *katG* S315T (100) | | | | | |
| G3-4 | 4.4.2 | Hr-TB | INH R | | *katG* S315T (100) | | | | | |
| G3-5 | 1.1.1.1 | Hr-TB | INH+STM R | | *katG* S315T (100) | | *gid* 102 del_G (100) | | | |
| G3-6 | 2.2.1 | Hr-TB | INH R | | *katG* S315T (100) | | | | | |
| G3-9 | 2.2.1 | Hr-TB | INH R | | *katG* S315T (100) | | | | | |
| G4-1 | 2.2.1 | Hr-TB | INH+STM+ETO R | | *katG* S315T (99) | | *rpsL* K43R (100) | *ethA* 639_640 del_GT (100) | | |
| G4-3 | 4.5 | Hr-TB | INH+STM R | | *katG* S315T (99) | | *rpsL* K88R (100) | | | |
| G4-4 | 2.2.1 | Hr-TB | INH+STM+ETO R | | *katG* S315T (99) | | *rpsL* K43R (100) | *ethA* 639_640 del_GT (99) | | |
| G4-6 | 1.2.1.2.1 | Hr-TB | INH+ETO R | | *fabG1* c-15t (100) | *inhA* I194T (99) | | *fabG1* c-15t (100) | *inhA* I194T (99) | |
| G5-4 | 2.2.1 | Hr-TB | INH+ETO R | | *fabG1* c-15t (98) | | | *fabG1* c-15t (98) | | |
| G5-5 | 1.1.3.3 | Hr-TB | INH+STM+ETO R | | *fabG1* c-15t (98) | | *gid* 102 del_G (99) | *fabG1* c-15t (98) | | |
| G6-1 | 1.1 | Hr-TB | INH+ETO R | | *inhA* g-154a (99) | | | *inhA* g-154a (99) | | |
| G6-2 | 4.5 | Sensitive | S | | | | | | | |

| MTB clinical isolate | MTB SNP-based sublineage | Drug resistance | Genotypic drug susceptibility | Rifampicin (% frequency ) | Isoniazid (% frequency) | | Streptomycin (% frequency) | Ethionamide (% frequency) | | Fluoroquinolone (% frequency) |
|---|---|---|---|---|---|---|---|---|---|---|
| G6-3 | 2.2.1 | Sensitive | S | | | | | | | |
| G6-4 | 1.2.1.2.1 | Hr-TB | INH+ETO R | | *fabG1* c-15t (98) | | | *fabG1* c-15t (98) | | |
| G6-5 | 4.4.2 | Sensitive | S | | | | | | | |
| G6-6 | 2.2.1 | Hr-TB | INH+ETO R | | *fabG1* c-15t (100) | | | *fabG1* c-15t (100) | | |
| G6-7 | 1.2.1.2.1 | Sensitive | S | | | | | | | |
| G6-8 | 1.1.3.3 | Hr-TB | INH+ETO R | | *fabG1* c-15t (100) | | | *fabG1* c-15t (100) | | |
| G6-9 | 2.2.1 | Other | STM R | | | | *rpsL* K88R (99) | | | |
| G6-10 | 2.2.1 | Hr-TB | INH R | | *katG* S315T (100) | | | | | |
| G6-11 | 2.2.1 | Hr-TB | INH+STM+ETO R | | *fabG1* c-15t (99) | | *rpsL* K88R (99) | *fabG1* c-15t (99) | | |
| G7-1 | 4.5 | Hr-TB | INH+ETO R | | *katG* S315T (100) | | | *ethA* 1265 del_C (100) | | |
| G7-2 | 2.2.1 | MDR-TB | RIF+INH R | *rpoB* L430P (100) | *katG* S315T (99) | | | | | |
| G7-3 | 2.2.1 | Hr-TB | INH R | | *katG* S315T (100) | | | | | |
| G7-4 | 2.2.1 | Hr-TB | INH+STM+ETO R | | *katG* S315T (99) | | *rpsL* K43R (100) | *ethA* 639_640 del_GT (99) | | |
| G7-7 | 2.2.1 | Hr-TB | INH R | | *katG* S315T (100) | | | | | |
| G7-8 | 2.2.1 | Hr-TB | INH R | | *katG* S315T (99) | | | | | |
| G7-9 | 1.1.3.3 | Hr-TB | INH+ETO R | | *fabG1* c-15t (100) | | | *fabG1* c-15t (100) | | |
| G7-10 | 1.2.1.2.1 | Hr-TB | INH+STM R | | *katG* S315T (100) | | *rpsL* K43R (100) | | | |
| G7-11 | 2.2.1 | Hr-TB | INH+STM R | | *katG* S315T (98) | *katG* A424G (99) | *rpsL* K43R (99) | | | |
| G8-1 | 1.1.1 | Sensitive | S | | | | | | | |
| G8-2 | 2.2.1 | Hr-TB | INH+STM+ETO R | | *inhA* g-154a (99) | | *rpsL* K43R (100) | *inhA* g-154a (99) | | |
| G8-3 | 2.2.1 | Other | STM R | | | | *rpsL* K43R (100) | | | |
| G8-4 | 4.5 | Sensitive | S | | | | | | | |
| G8-5 | 2.2.1 | Hr-TB | INH+STM+ETO R | | *fabG1* c-15t (99) | | *rpsL* K43R (99) | *fabG1* c-15t (99) | | |
| G8-6 | 1.2.1.2.1 | Hr-TB | INH+ETO R | | *fabG1* c-15t (100) | | | *fabG1* c-15t (100) | | |
| G8-8 | 1.2.1.2.1 | Hr-TB | INH+ETO R | | *fabG1* c-15t (99) | | | *fabG1* c-15t (99) | | |
| G8-12 | 1.1.1 | Hr-TB | INH+ETO R | | *fabG1* c-15t (99) | | | *fabG1* c-15t (99) | | |
| G8-15 | 1.1.1 | Sensitive | S | | | | | | | |
| G8-16 | 1.2.1.2.1 | Sensitive | S | | | | | | | |

| MTB clinical isolate | MTB SNP-based sublineage | Drug resistance | Genotypic drug susceptibility | Rifampicin (% frequency ) | Isoniazid (% frequency) | | Streptomycin (% frequency) | Ethionamide (% frequency) | | Fluoroquinolone (% frequency) |
|---|---|---|---|---|---|---|---|---|---|---|
| G8-17 | 1.2.1.2.1 | Hr-TB | INH+ETO R | | *fabG1* c-15t (100) | | | *fabG1* c-15t (100) | | |
| G8-18 | 1.2.1.2.1 | Hr-TB | INH+ETO R | | *fabG1* c-15t (99) | | | *fabG1* c-15t (99) | | |
| G8-19 | 1.1.1.1 | Hr-TB | INH+ETO R | | *fabG1* c-15t (100) | | | *fabG1* c-15t (100) | | |
| G8-20 | 1.2.1.2 | Hr-TB | INH+ETO R | | *inhA* g-154a (100) | | | *inhA* g-154a (100) | | |

[a] Mutations detected by WGS with frequency of variants occurring in 98% to 100% of reads.

[b] WGS-based gDST, whole-genome sequencing-based genotypic drug susceptibility testing; MTB, *Mycobacterium tuberculosis*; No., number; RIF, rifampicin; INH, isoniazid; FLQ, fluoroquinolone; STM, streptomycin; ETO, ethionamide; R, resistant; S, susceptible; MDR-TB; multidrug-resistant tuberculosis; Hr-TB, isoniazid-resistant tuberculosis.

**Table S3 Comparison of WGS-based gDST and MGIT-based pDST [a]**

| Drug | WGS | MGIT | | % (No.) of false negative results | % (No.) of false positive results | PPV (%) | NPV (%) | % Categorical agreement (95% CI) |
|---|---|---|---|---|---|---|---|---|
| | | R | S | | | | | |
| INH | R | 45 | 0 | 19.6 (11/56) | 0 (0/0) | 100.0 | n/a | 80.4 (67.2 - 89.3) |
| | S | 11 | 0 | | | | | |
| RIF | R | 0 | 3 | 0 (0/0) | 5.3 (3/56) | n/a | 100.0 | 94.6 (84.2 - 98.6) |
| | S | 0 | 53 | | | | | |
| EMB | R | 0 | 0 | 100.0 (1/1) | 0 (0/55) | n/a | 98.2 | 98.2 (89.2 - 99.9) |
| | S | 1 | 55 | | | | | |
| PZA | R | 0 | 0 | 100.0 (15/15) | 0 (0/41) | n/a | 73.2 | 73.2 (59.5 - 83.8) |
| | S | 15 | 41 | | | | | |
| LFX | R | 1 | 0 | 50.0 (1/2) | 0 (0/54) | 100.0 | 98.2 | 98.2 (89.2 - 99.9) |
| | S | 1 | 54 | | | | | |
| STM | R | 11 | 3 | 35.3 (6/17) | 7.7 (3/39) | 78.6 | 85.7 | 83.9 (71.2 - 92.0) |
| | S | 6 | 36 | | | | | |

For this comparison, MGIT was considered the reference standard. A false negative result indicates that MGIT, but not WGS-based gDST, detected drug resistance. A false positive result indicates that WGS-based gDST, but not MGIT, detected drug resistance.

[a] WGS-based gDST, whole-genome sequencing-based genotypic drug susceptibility testing; pDST, phenotypic drug susceptibility testing; WGS, whole-genome sequencing; MGIT, mycobacterium growth indicator tube; No., number; RIF, rifampicin; INH, isoniazid; EMB, ethambutol; PZA, pyrazinamide; LFX, levofloxacin; STM, streptomycin; PPV, positive predictive value; NPV, negative predictive value; 95% CI, 95% confidence interval; R, resistant; S, susceptible; n/a, not applicable.

## Table S4 Comparison of WGS-based gDST and BMD-based pDST [b]

| Drug [a] | WGS | BMD | | % (No.) of false negative results | % (No.) of false positive results | PPV (%) | NPV (%) | % Categorical agreement (95% CI) |
|---|---|---|---|---|---|---|---|---|
| | | R | S | | | | | |
| INH | R | 44 | 1 | 17.0 (9/53) | 33.3 (1/3) | 97.8 | 18.2 | 82.1 (69.2 - 90.7) |
| | S | 9 | 2 | | | | | |
| RIF | R | 1 | 2 | 0 (0/1) | 3.6 (2/55) | 33.3 | 100.0 | 96.4 (86.6 - 99.4) |
| | S | 0 | 53 | | | | | |
| EMB | R | 0 | 0 | 0 (0/0) | 0 (0/56) | n/a | 100.0 | 100.0 (n/a) |
| | S | 0 | 56 | | | | | |
| LFX | R | 0 | 1 | 0 (0/0) | 1.8 (1/56) | n/a | 100.0 | 98.2 (89.2 - 99.0) |
| | S | 0 | 55 | | | | | |
| MFX | R | 0 | 1 | 0 (0/0) | 1.8 (1/56) | n/a | 100.0 | 98.2 (89.2 - 99.0) |
| | S | 0 | 55 | | | | | |
| BDQ | R | 0 | 0 | 0 (0/0) | 0 (0/56) | n/a | 100.0 | 100.0 (n/a) |
| | S | 0 | 56 | | | | | |
| LZD | R | 0 | 0 | 0 (0/0) | 0 (0/56) | n/a | 100.0 | 100.0 (n/a) |
| | S | 0 | 56 | | | | | |
| CFZ | R | 0 | 0 | 0 (0/0) | 0 (0/56) | n/a | 100.0 | 100.0 (n/a) |
| | S | 0 | 56 | | | | | |
| KAN | R | 0 | 0 | 100.0 (1/1) | 0 (0/55) | n/a | 98.2 | 98.2 (89.2 - 99.0) |
| | S | 1 | 55 | | | | | |
| AMK | R | 0 | 0 | 0 (0/0) | 0 (0/56) | n/a | 100.0 | 100.0 (n/a) |
| | S | 0 | 56 | | | | | |
| ETO | R | 16 | 8 | 20.0 (4/20) | 22.2 (8/36) | 66.7 | 87.5 | 78.6 (65.2 - 88.0) |
| | S | 4 | 28 | | | | | |

For this comparison, BMD was considered the reference standard. The MIC interpretation to S or R categories was based on the CRyPTIC ECOFF/ECVs (1). A false negative result indicates that MGIT, but not WGS-based gDST, detected drug resistance. A false positive result indicates that WGS-based gDST detected drug resistance, but MGIT revealed susceptibility.

[a] CAP was excluded from this analysis because no interpretative breakpoint was addressed by WHO and CRyPTIC.
[b] WGS-based gDST, whole-genome sequencing-based genotypic drug susceptibility testing; pDST, phenotypic drug susceptibility testing; WGS, whole-genome sequencing; BMD, broth microdilution; No., number; RIF, rifampicin; INH, isoniazid; EMB, ethambutol; PZA, pyrazinamide; LFX, levofloxacin; MFX, moxifloxacin; BDQ, bedaquiline; LZD, linezolid; CFZ, clofazimine; KAN, kanamycin; AMK, amikacin; ETO, ethionamide; CAP, capreomycin; PPV, positive predictive value; NPV, negative predictive value; 95% CI, 95% confidence interval; R, resistant; S, susceptible; n/a, not applicable.

**Reference**

1. The CRyPTIC Consortium. 2022. Epidemiological cut-off values for a 96-well broth microdilution plate for high-throughput research antibiotic susceptibility testing of *M. tuberculosis*. Eur Respir J 60:2200239.

## Table S5 WGS-based gDST results and MIC values of the 56 INH-resistant MTB clinical isolates [d]

| MTB clinical isolate | MTB SNP-based sublineage | WHO Confidence grading of mutations (% frequency) [a] | | | | INH MIC (mg/L) |
|---|---|---|---|---|---|---|
| | | Assoc w INH R | | Not Assoc w INH R | | |
| G1-1 | 2.2.1 | *katG* S315T (100) | | *katG* R463L (100) | *rv1258c* 580_581insC (100) | 2 |
| G1-2 | 2.2.1 | *katG* S315T (100) | | *katG* R463L (100) | | 2 |
| G1-3 | 2.2.1 | | | *katG* R463L (100) | *katG* P422L (100) [b] | 2 |
| G1-4 | 2.2.1 | *katG* S315T (100) | | *katG* R463L (100) | *rv1258c* 580_581insC (100) | 4 |
| G2-1 | 1.2.1.2 | *katG* S315T (100) | | *katG* R463L (99) | | 4 |
| G2-4 | 2.2.1 | *katG* S315T (100) | | *katG* R463L (100) | *rv1258c* 580_581insC (100) | 2 |
| G2-5 | 1.1.3.3 | *katG* S315T (100) | *fabG1* c-15t (99) | *katG* R463L (100) | | >16 |
| G2-6 | 1.2.1.2.1 | *katG* S315T (99) | | *katG* R463L (100) | *fabG1* t-8g (99) [b] | 8 |
| G2-7 | 1.1.1 | *katG* S315T (100) | | *katG* R463L (100) | | 4 |
| G3-1 | 2.2.1 | *katG* S315T (100) | | *katG* R463L (100) | *rv1258c* 580_581insC (100) | 2 |
| G3-2 | 2.2.1 | *katG* S315T (100) | | *katG* R463L (100) | *rv1258c* 580_581insC (100) | 4 |
| G3-3 | 2.2.1 | *katG* S315T (100) | | *katG* R463L (100) | *rv1258c* 580_581insC (100) | 2 |
| G3-4 | 4.4.2 | *katG* S315T (100) | | | | 4 |
| G3-5 | 1.1.1.1 | *katG* S315T (100) | | *katG* R463L (100) | | 4 |
| G3-6 | 2.2.1 | *katG* S315T (100) | | *katG* R463L (100) | *rv1258c* 580_581insC (100) | 4 |
| G3-9 | 2.2.1 | *katG* S315T (100) | | *katG* R463L (99) | *rv1258c* 580_581insC (100) | 4 |
| G4-1 | 2.2.1 | *katG* S315T (99) | | *katG* R463L (100) | *rv1258c* 580_581insC (100) | 4 |
| G4-3 | 4.5 | *katG* S315T (99) | | *katG* R463L (100) | | 2 |
| G4-4 | 2.2.1 | *katG* S315T (99) | | *katG* R463L (100) | *rv1258c* 580_581insC (100) | 2 |
| G4-6 | 1.2.1.2.1 | *fabG1* c-15t (100) | | *katG* R463L (100) | *inhA* I194T (99) [b] | 2 |
| G5-4 | 2.2.1 | *fabG1* c-15t (98) | | *katG* R463L (100) | *rv1258c* 580_581insC (100) | 0.25 |
| G5-5 | 1.1.3.3 | *fabG1* c-15t (98) | | *katG* R463L (100) | | 0.25 |
| G6-1 | 1.1 | *inhA* g-154a (99) | | *katG* R463L (100) | | 0.25 |
| G6-2 | 4.5 | | | | | 0.25 |
| G6-3 | 2.2.1 | | | *katG* R463L (100) | *rv1258c* 580_581insC (100) | 0.25 |
| G6-4 | 1.2.1.2.1 | *fabG1* c-15t (98) | | | | 0.25 |

| MTB clinical isolate | MTB SNP-based sublineage | WHO Confidence grading of mutations (% frequency) [a] | | | | INH MIC (mg/L) |
|---|---|---|---|---|---|---|
| | | Assoc w INH R | | Not Assoc w INH R | | |
| G6-5 | 4.4.2 | | | | | 0.25 |
| G6-6 | 2.2.1 | *fabG1* c-15t (100) | | *katG* R463L (100) | *rv1258c* 580_581insC (100) | 0.25 |
| G6-7 | 1.2.1.2.1 | | | *katG* R463L (100) | | 0.25 |
| G6-8 | 1.1.3.3 | *fabG1* c-15t (100) | | *katG* R463L (100) | | 0.25 |
| G6-9 | 2.2.1 | | | *katG* R463L (100) | *rv1258c* 580_581insC (100) | 0.25 |
| G6-10 | 2.2.1 | *katG* S315T (100) | | *katG* R463L (100) | *rv1258c* 580_581insC (100) | 2 |
| G6-11 | 2.2.1 | *fabG1* c-15t (99) | | | | 2 |
| G7-1 | 4.5 | *katG* S315T (100) | | | | 2 |
| G7-2 | 2.2.1 | *katG* S315T (99) | | *katG* R463L (100) | *rv1258c* 580_581insC (100) | 1 |
| G7-3 | 2.2.1 | *katG* S315T (100) | | *katG* R463L (100) | *rv1258c* 580_581insC (100) | 1 |
| G7-4 | 2.2.1 | *katG* S315T (99) | | *katG* R463L (100) | *rv1258c* 580_581insC (100) | 2 |
| G7-7 | 2.2.1 | *katG* S315T (100) | | *katG* R463L (100) | *rv1258c* 580_581insC (100) | 2 |
| G7-8 | 2.2.1 | *katG* S315T (99) | | *katG* R463L (100) | *rv1258c* 580_581insC (100) | 2 |
| G7-9 | 1.1.3.3 | *fabG1* c-15t (100) | | *katG* R463L (100) | | 0.5 |
| G7-10 | 1.2.1.2.1 | *katG* S315T (100) | | | | 0.5 |
| G7-11 | 2.2.1 | *katG* S315T (98) | | *katG* R463L (100) | *rv1258c* 580_581insC (100) | 2 |
| | | | | | *katG* A424G (99) [b] | |
| G8-1 | 1.1.1 | | | *katG* R463L (100) | | 0.25 |
| G8-2 | 2.2.1 | *inhA* g-154a (99) | | *katG* R463L (100) | *rv1258c* 580_581insC (100) | 0.25 |
| G8-3 | 2.2.1 | | | *katG* R463L (100) | *rv1258c* 580_581insC (100) | 0.25 |
| G8-4 | 4.5 | | | | | 0.12 |
| G8-5 | 2.2.1 | *fabG1* c-15t (99) | | *katG* R463L (100) | *rv1258c* 580_581insC (100) | 0.25 |
| G8-6 | 1.2.1.2.1 | *fabG1* c-15t (100) | | *katG* R463L (100) | | 0.12 |
| G8-8 | 1.2.1.2.1 | *fabG1* c-15t (99) | | *katG* R463L (100) | | 0.25 |
| G8-12 | 1.1.1 | *fabG1* c-15t (99) | | *katG* R463L (100) | | 0.5 |
| G8-15 | 1.1.1 | | | *katG* R463L (100) | | 0.12 |
| G8-16 | 1.2.1.2.1 | | | *katG* R463L (100) | | 0.25 |
| G8-17 | 1.2.1.2.1 | *fabG1* c-15t (100) | | *katG* R463L (100) | | 0.25 |

| MTB clinical isolate | MTB SNP-based sublineage | WHO Confidence grading of mutations (% frequency) [a] | | | | INH MIC (mg/L) |
|---|---|---|---|---|---|---|
| | | Assoc w INH R | | Not Assoc w INH R | | |
| G8-18 | 1.2.1.2.1 | *fabG1* c-15t (99) | | *katG* R463L (100) | | 0.25 |
| G8-19 | 1.1.1.1 | *fabG1* c-15t (100) | | *katG* R463L (100) | *katG* g-278c (99) [c] | 0.25 |
| G8-20 | 1.2.1.2 | *inhA* g-154a (100) | | *katG* R463L (100) | | 0.25 |

[a] Blanks indicate that none of the mutations related to INH susceptibility were detected.
[b] Mutation is not listed in the WHO catalogue of MTBC mutations (1).
[c] Final confidence grading from the WHO catalogue of MTBC mutations is "Not associated with resistance-interim" for INH (1).
[d] WGS-based gDST, whole-genome sequencing-based genotypic drug susceptibility testing; MTB, *Mycobacterium tuberculosis*; INH, isoniazid; MIC, minimal inhibitory concentration; Assoc w INH R, associated with isoniazid resistance; Not Assoc w R, not associated with isoniazid resistance.

**Reference**


1. WHO. 2021. Catalogue of mutations in *Mycobacterium tuberculosis* complex and their association with drug resistance. Licence: CC BY-NCSA 3.0 IGO. Geneva: World Health Organization.

**Table S6 Mutations detected in PZA-susceptible and PZA-resistant MTB isolates [c]**

| MTB clinical isolate | | Mutations and no. of isolates | | | | | | | | |
|---|---|---|---|---|---|---|---|---|---|---|
| | | *pncA* | *clpC1* | | *panD1* | *rv1258c* | PPE35 | | | *rv3236c* |
| **PZA MGIT result** | **No.** | | **V63A [a]** | **P796L [a]** | | **G194fs [b]** | **G877A [b]** | **L896S [a]** | **I15M [b]** | **T102A [a]** |
| **S** | 41 | - | 15 | 3 | - | 21 | 5 | 37 | 3 | 22 |
| **R** | 15 | - | 7 | 1 | - | 5 | 6 | 14 | 1 | 5 |

[a] Final confidence grading from the WHO catalogue of MTBC mutations is not associated with PZA resistance (1).
[b] Mutation is not listed in the WHO catalogue of MTBC mutations (1).
[c] MTB, *Mycobacterium tuberculosis*; PZA, pyrazinamide; no., number; MGIT, Mycobacterium Growth Indicator Tube; R, resistant; S, susceptible.

## Reference

1. WHO. 2021. Catalogue of mutations in *Mycobacterium tuberculosis* complex and their association with drug resistance. Licence: CC BY-NCSA 3.0 IGO. Geneva: World Health Organization.
